# Evidential value of country location evidence obtained from IP address geolocation

**DOI:** 10.7717/peerj-cs.1305

**Published:** 2023-03-30

**Authors:** Dan Komosny

**Affiliations:** Department of Telecommunications, FEEC, Brno University of Technology, Brno, Czech Republic

**Keywords:** Internet, IP address, Service, Location, Country, Evidence, Forensics

## Abstract

Knowledge of the previous location of an Internet device is valuable information in forensics. The previous device location can be obtained via the IP address that the device used to access Internet services, such as email, banking, and online shopping. However, the problem with the device location using its IP address is the unknown evidential value, which is used to admit the evidence in the case. This work introduces a method to process free and constantly updated data to assess the evidential value of the IP country location. The evidential value is assessed for several countries by analyzing historical data over 8 years. Tampering with the location evidence is discussed, as well as its detection. The source code to replicate the results and to apply the updated data to future evidence is available.

## Introduction

Knowledge of the previous location of a person is important in crime investigation. Direct location evidence is the testimony of a witness about seeing someone in a place. Indirect evidence in terms of digital forensics may be the location of the device that was used by the person at the given time. The location evidence has an assigned evidential value. This evidential value is used to admit the evidence in the case, Imhoff (2020) and Jury Instructions Committee (2022).

Internet devices communicate through unique public IP addresses. It is possible to estimate the location of the public IP address, and thus the location of the device. IP address geolocation is a universal solution without the need to have access to the device and is independent of the device hardware and software. It also allows for exploring past device locations, provided that the device’s previous IP addresses are known. Therefore, (theoretically) IP address geolocation is a great tool in forensics. However, the accuracy of IP geolocation varies greatly. It varies between places and changes over time. Such differences are caused by constant movements in the IP address space and other limitations, such as different population densities.

**Figure 1:**
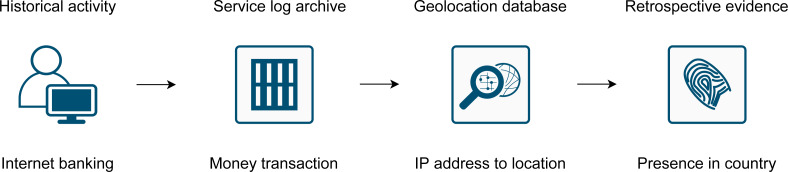
Process to obtain indirect evidence of a person’s previous location in a country. Certain assumptions are a prerequisite.

The previous locations of a device (person) can be obtained *via* online services the person accessed. Services commonly archive user activity in their logs, as shown in Fig. 1. The log typically consists of the person’s username and the IP address of the device that was used to access the service. Inferring that the person uses her/his username, the location of the person can be obtained from the device IP address. It should be noted that the credentials (username, password) for a personal account can be shared, and this would result in false evidence. This situation is analogous to the tracking of a mobile phone when it is passed on to another person or left alone. This possibility is discussed further.

The strength of IP geolocation use in forensics is given by the multiple data sources available. A typical person uses many online services, which keep track of the person’s activity. Table 1 lists the services whose activity log can be used to obtain location evidence. The services are sorted according to the empirically assumed access frequency. Services that are accessed more frequently are more likely to provide location evidence. The service account must be firmly linked to the person so that it can be used as evidence against the person. For example, a person is firmly linked to the service account by an authority. These services are work (intranet) where the authority is the employer, education (elearning) where the authority is the university, and banking where the authority is the bank. A person needs to prove ownership of the service account to use location evidence as the alibi, *e.g.*, that the account was breached. Ownership of the service can be proven by knowing the password and the full name associated with the account. A special case is online shopping, where the person provides the name and address for the delivery. Account credentials can be shared, or a VPN service can be used to create forged evidence. It is further shown that this can be potentially detected by combining multiple data sources.

**Table 1:** List of services whose logs can provide location evidence. Frequency shows (empirically) how often the service is used.

Freq.	Service	Description
High	Email	Email client periodically checks (e.g., every 10 min) for new messages
High	Chat	Chat client keeps long-lived connections for instant message delivery
Med	Social	Person has to login to publish personal data
Med	Work	Person has to log into the company intranet to access work data
Low	Education	Student has to login to access course data, curricula, and exams
Low	E-shop	Person provides full name for goods delivery
Low	Banking	Person has to log into the bank site to manage the accounts

The availability of the service logs is given by the fact that the service providers and organizations commonly archive the users’ activity for their own profit. The reasons include marketing (e-shop, chat, social), statistics (education), and security (email, work, banking). The services indicated in parentheses are not exclusive. For example, universities commonly archive students’ activity for various analyses, including students’ online examination performance, Yousef & Sumner (2021) and Moodle (2022). Logs can also be used to detect student cheating during online exams. It depends on the law of the country, whether the service logs are available for forensic purposes, Casey (2011). A service provider or organization may also provide the data for investigation on a voluntary basis.

This work attempts to solve the problem of IP geolocation use in forensics. Each digital evidence should have an evidential value assigned. This value is considered by the court to admit the evidence in the case. For IP geolocation, the evidential value may include the location accuracy, volume of the ground-truth data, population and geographical layout of the country, and accuracy changes over time. However, the evidential value of IP geolocation is different per place. For these reasons, each location evidence should have its evidential value assessed individually. In the case of country location, the evidential value should be assessed for the specific country. This work shows a way to process free and constantly updated data to assess the evidential value of IP country location. The presented solution is applicable to IP addresses of broadband devices. This work is not applicable to cellular devices.

The article is structured as follows. The first section deals with the problem of IP geolocation use in forensics. The contribution of this work is also described. The section ‘Related work’ describes the current state of IP geolocation with a focus on the country level. The section ‘Solution to assess evidential value’ introduces a way of processing free data to assess the evidential value of IP country location. The evidential value for several countries is presented in the next section. The use of this work in forensics is discussed in the sections ‘Tampered evidence’ and ‘Method and data validity’.

## Problem and Contribution

The problems of assessing the evidential value of IP country location are elaborated first. The benefits of the solution are highlighted next. The problems are as follows:

(i) IP geolocation accuracy changes over time. It cannot be assumed that the accuracy known *via* a single measurement a year ago is applicable to the current time. Nor that a one-time measurement processed just before the location evidence date is a good representative of the accuracy, as a peak value could be observed. The reason is that the IP address space is constantly changing, Mouram et al. (2015) and Xu, Fujikawa & Harai (2013). The reallocation of IP addresses at international ISPs is particularly important.

(ii) Countries differ in ICT (Information and Communication Technologies) levels, population density, and geography. It cannot be said that the accuracy in one country is the same as in another. There are many technical aspects related to ICT, such as the number of international ISPs, the size of the IP address space, and the number of peering points. The geography of the country is also important. In particular, the presence of exclaves/enclaves and the number of neighboring countries (*e.g.*, sealocked country *vs.* inland country) affect the IP geolocation accuracy at the country level. Population density plays a role when personal devices are used to source geolocation data (*e.g.*, smartphones that report their location). Differences between countries are well shown in IP2Location (2022a), BigDataCloud (2022b) and MaxMind (2022).

(iii) Ground-truth data, that is, IP addresses with known correct location, are not generally accessible for forensic purposes. Previous works dealing with the accuracy of IP geolocation were based on private data, or public data without the updates available, Callejo et al. (2022), BigDataCloud (2022a), Gharaibeh et al. (2017) and Livadariu et al. (2020). Therefore, these ground-truth data cannot be used to assess the evidential value of IP location evidence.

The contribution of this work is a transparent assessment of the evidential value of IP country location. The presented evidential method is universal and is applicable to past and future evidence. The problem of accuracy changes over time is addressed by the idea of employing historical data, which solves the problem no. 1. Firm and stable results are particularly required in forensics. The problem of country differences in location accuracy is addressed by using historical data that are specific for each country. This addresses the problem no. 2. The evidential method is based on free and constantly updated data. In this way, the process of establishing the evidential value is reproducible and applicable to future evidence. The use of free data addresses the problem no. 3.

## Related Work

Research in digital forensics covers various sources of information, including network communication and data stored in databases. Evidence of the presence of the device (user) in a country involves two data sources, which are the device IP address and user activity stored in a database. The use of IP addresses in forensics was elaborated in Koch et al. (2016). The work dealt with setting up an environment that allows forensic investigations of cyber attacks. The proposal covered the extension of the logging system using the information from IP geolocation. It was suggested that more information is archived for devices that come from known suspicious locations, including routing data, IP address ranges, and ISP changes. Mirza & Karabiyik (2021) elaborated the forensic information that an IP address can reveal. The article focused on forensic tools and discussed the lack of data related to IP addresses. An IP visualization technique was proposed to merge several sources for digital evidence. The speed of forensic investigation was reduced by the developed tool. IP addresses are also used to prevent different types of fraud. Gulati et al. (2017) combined IP address geolocation with machine learning to detect suspicious credit card payments. The cardholder pattern of spending and managing money was used as input for machine learning.

IP addresses can provide an approximate location of the device, which is useful in forensics. On the other hand, a person’s privacy can be breached, for example, when applications reporting IP addresses and GPS coordinates are used on smartphones, Keßler & McKenzie (2018). Measures to preserve user location privacy in mobile applications were studied by Liu et al. (2018). The authors dealt with various cases, such as user identity, proximity information to nearby places, and travel trajectories. Attacks were also elaborated to breach user location privacy, and possible solutions were given. The solutions included hiding the user location through a privacy server (*e.g.*, by k-anonymization) and obfuscation of the correlated locations.

The use of IP addresses in forensics depends on the level of location accuracy. The accuracy at the country level was elaborated by Gharaibeh et al. (2017). The work focused on the location of IP addresses that belonged to routers. Commercial and free geolocation databases were used. The free geolocation databases were IP2Location DB11 LITE and MaxMind GeoLite2. The commercial databases were MaxMind GeoIP2 and Digital Envoy NetAcuity. The ground-truth dataset consisted of around 17,000 IP addresses. This dataset was created using two methods. The first was based on decoding the locations from the router hostnames, which include hints of their physical location (*e.g.*, city names and their abbreviated forms). The second method was based on the RIPE Atlas. The Atlas provides periodic traceroute measurements from Atlas devices to a set of targets, including DNS root servers. The IP addresses of the routers on the paths were collected. Latency values between routers and Atlas devices lower than 0.5 ms were used to locate routers that were within a 50 km distance from Atlas devices. The known location of the Atlas devices was assigned to the router IP addresses. The result was given in the form of country-level accuracy for 20 countries. There were large differences in accuracy between the countries. For the US and Russia, all databases had a country-level accuracy better than 94%. For Germany and Great Britain, the accuracy was around 60%, except the NetAcuity database, which showed an accuracy of around 80%. The accuracy in Italy was around 90% for all databases. The NetAcuity database provided the best overall accuracy, in some countries twice the accuracy of other databases, such as in France and Canada. There were also differences between the databases in the geographical regions defined by IANA. For example, the addresses associated with RIPE NCC (Europe, Middle East and Central Asia) and ARIN (US, Canada, and other countries) were located in the correct country by the IP2Location LITE database with around 77% accuracy. The MaxMind GeoLite2 database located the IP addresses of RIPE NCC and ARIN in the correct country with around 70.5% and 78.9% accuracy, respectively. The collected ground truth is publicly provided.

The country-level geolocation accuracy of cellular IP addresses was analyzed by Callejo et al. (2022). The IP addresses located belonged to smartphones and tablets, which could also be connected *via* WiFi with broadband access. Each device had an application installed with a particular SDK (Software Development Kit). The applications provided various free services, such as weather forecasts and news. The applications sent the location of the device (obtained *via* GPS) and its IP address to the SDK provider, provided that permission was granted. IP addresses and their locations were collected for one month. On average, 22 million locations were collected in Spain, France, and Great Britain. Two IP geolocation databases were used; however, their names were not given. The name of the SDK provider that collects the locations was also not given. The authors justified the anonymity of the databases not to compare them, but to evaluate the use of IP geolocation in online advertising. The results showed that the country-level accuracy of the two databases was 100% when rounded. The accuracy decreased with the region and city levels. Large differences were observed between the countries. The accuracy at smaller levels was shown to be strongly correlated with the density of the population. In Spain, the accuracy was approximately 75% in urban and 50% in rural areas, defined as autonomous communities. In France, the values were approximately 80 and 65% in urban and rural areas (respectively), defined as regions. In Great Britain, the accuracy was approximately 70 and 50% in urban and rural areas (respectively), defined as counties. The ground truth is not publicly accessible.

BigDataCloud (2022a), an IP geolocation provider, published a report that includes accuracy at the country level. The ground-truth data come from three sources: (i) provider’s website visitors. Visitors are asked to share their location, which is linked to their IP address. (ii) Mobile device users that use free applications, such as networking tools. Users are asked to share their GPS location in exchange for using the application, provided that permission is granted. This location is associated with the device IP address. (iii) General users who use the reverse geocoding API. The client-side policy assumed that the GPS location requested to be translated to an address is the location of the device and its IP address. Around 900,000 IPv4 addresses were used in the daily report. The ground truth included about 440,000 wired device addresses and about 375,000 cellular addresses. The rest of the addresses belonged to hosting and mixed network devices. The results were shown for several geolocation databases and services: IP2Location DB5, MaxMind GeoLite2, DB-IP, WhoisXmlApi, and Big Data Cloud. The country-level accuracy was around 99% for all providers. The results showed only small differences for the wired and cellular IP addresses. A larger accuracy drop was observed for the hosting and mixed network devices, which were correctly located in a country with around 97% accuracy. The results were also given for specific countries. The accuracy for IP addresses of all device types was around 98% in Germany. There was a large gap between the wired and cellular IP addresses, where the accuracy was around 99 and 94%, respectively. The ground truth for Germany included about 10,000 IP addresses. Of them, 7,000 belonged to wired devices and 2,000 belonged to cellular devices. The accuracy ranged from 92 to 96% for all types of devices in Spain. The IP addresses of the wired and cellular devices were correctly located with an accuracy from 98 to 99%, and from 84 to 89% (respectively), which was a larger difference than in Germany. The number of ground-truth IP addresses in Spain was 14,000. Of them, 9,000 belonged to wired devices and 5,000 belonged to cellular devices. The ground truth is not publicly accessible.

IP2Location (2022a) published the accuracy in countries for a radius of 50 miles. For example, the accuracy was around 70% in Turkey and 87% in Finland. The number of ground-truth IP addresses was not given, only their range of hundreds to thousands per country. The type of addresses (broadband, cellular) was also not given. Ground-truth data are not publicly accessible. A similar type of result was given in MaxMind (2022). The accuracy was reported for three versions of the MaxMind databases and services: GeoLite2 City (free), GeoIP2 City (commercial), and GeoIP2 City Plus web service (commercial). There was a large difference between the countries. For example, 87% of broadband addresses were correctly located within a radius of 250 km in Spain, compared to only 44% of cellular addresses. The accuracy at the same level was 95% for the broadband addresses in Germany, and 59% for the cellular addresses. The results are given for the GeoIP2 City Plus web service. The free database GeoLite2 City showed 3% lower accuracy within the 250 km radius in Spain and 1% lower accuracy in Germany for broadband addresses. The accuracy drop was larger for smaller radii: 7% for the city level in Spain and 5% in Germany. The number of IP addresses used and ground-truth data are not publicly accessible.

In conclusion, the related work shows differences in the accuracy of IP geolocation. Differences are observed in countries and for broadband and cellular IP addresses. Geolocation databases have a similar accuracy at the country level for the broadband IP addresses of end devices. However, geolocation databases differ when IP addresses of routers are located. The location accuracy is significantly lower than for end-device IP addresses.

## Solution to Assess Evidential Value

**Figure 2:**
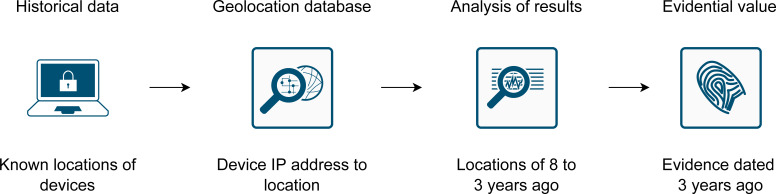
Basic overview to assess the evidential value of IP country location.

The solution allows for a transparent assessment of the evidential value of IP country location. The basic overview is shown in Fig. 2. The accuracy of IP geolocation is dynamic due to constant movements in the IP address space. Therefore, historical data are used to observe changes in accuracy over time. The historical data used are free and continually updated. This allows us to assess the evidential value of past and future evidence. The historical data are also country specific, as the global accuracy cannot be applied in forensics, nor the accuracy of a country to another country.

The historical data are formed by tuples that include the IP address, the date of its use by the device, and the location of the device in a country. To obtain these elements, the source data are processed and historically linked. Processing of the source data is described in the first subsection. Their validation is described in the second subsection.

### Source data processing

**Figure 3:**
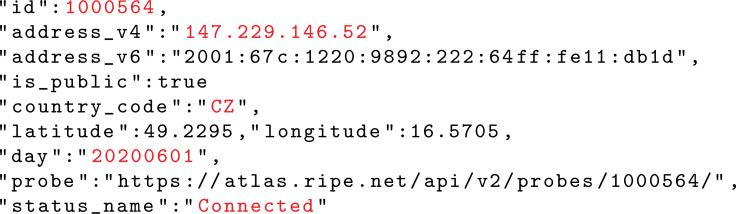
Selected elements of the Atlas device operation report dated June 1st 2020. Highlighted items are important for the solution.

RIPE Atlas is used as a data source, RIPE NCC (2022b). The atlas consists of a large set of end devices that are distributed worldwide. Device operational data are continuously collected and archived. The data are archived in the form of daily reports. Figure 3 lists a set of items stored in a daily report. The elements that are important for the method are the device ID, IP address, country code, date, and operational status. The operational status of a device can be ‘Connected’, ‘Abandoned’, ‘Disconnected’, and ‘Never Connected’. Devices with the ‘Connected’ status were alive on the given day and were required to actively communicate with an Atlas controller.

The daily reports are filtered as follows: (i) devices with other statuses than ‘Connected’ are excluded as their IP addresses may have been outdated or already reallocated on the past day, (ii) devices with an unknown latitude/longitude or country are excluded, and (iii) devices with coordinates and country mismatch are excluded. All IP addresses are validated to be unique global and the others are removed.

### Historical dataset compilation

The historical dataset is compiled by linking the operational data of the devices. The data are further validated. The linking process covers filtering IP addresses that were assigned to a device over a row of days. The presence of repetitive addresses would provide inaccurate results, which is not acceptable in forensics. For example, if a device used the same address in a row of 100 days, the same correct/wrong country location would be counted 100 times. On the other hand, an IP address that was used for one day would be counted only once. For this reason, only the most distant device IP addresses are used. The unique ID of the Atlas device (shown in Fig. 3) is used to implement IP address filtering. Table 2 shows an example. The IP addresses in the table were used by a device in a row of days. The addresses on the most distant dates are highlighted.

**Table 2:** IP addresses used by a device. IP addresses that are used in the solution are highlighted.

37.221.246.123	37.221.246.123	...	37.221.246.123	37.221.246.116	37.221.246.116	...	37.221.246.116	95.82.149.70	95.82.149.70	...	95.82.149.70	46.39.182.135	46.39.182.135	...	46.39.182.135	83.240.4.221	83.240.4.221	...	83.240.4.221
older past day —————————————————————————————————————–> newer past day																			

The historical dataset is further validated for consistency. There were several devices whose country changed over time. These devices were excluded from the entire historical range since their country was uncertain. There are also IP addresses that are not suitable for location accuracy evaluation, Lewis et al. (2020). These IP addresses are not within the on-premise address ranges. Typically, the IP addresses of the devices connected to the cloud are not in the ISP or organization address range, Almohri, Watson & Evans (2020). Cloud service providers provide a limited range of IP addresses. Therefore, they may frequently reallocate the addresses to the devices, Padmanabhan et al. (2016). Table 3 shows examples of such reallocation. The table includes IP addresses that belonged to three cloud service providers. All listed IP addresses were geolocated to the wrong country, that is, to the country that is different from the true country of the device. In the first example, the device true country is Great Britain, but the device IP addresses were located to Belgium. These IP addresses are shown to change frequently. The second example shows the addresses that belonged to a device in China. These IP addresses pointed to a different country (South Korea) and also to a different continent (US). The last example shows IP addresses that were assigned to a device in Ireland. The addresses pointed to multiple countries, including Germany, Canada, and the Netherlands. The cloud IP addresses of Amazon Web Services, Google Cloud Platform, Digital Ocean, and Microsoft Azure are filtered from the historical dataset. It should be noted that this filtering is not comprehensive and that some cloud addresses may still be present in the dataset.

**Table 3:** Examples of IP addresses that are not suitable for evaluating the location accuracy. All IP address locations were wrong. Example with Google Cloud Platform–device IP addresses changed frequently. Example with Amazon Web Services–device IP addresses pointed to different continents. Example with Digital Ocean–device IP addresses pointed to a number of countries.

Cloud service	ASN	IP address	Date	Device country	IP country
Google Cloud Platform	396982	34.78.21.28	2022-12-23	GB	BE
Google Cloud Platform	396982	35.195.40.235	2022-12-12	GB	BE
Google Cloud Platform	396982	104.199.50.15	2022-12-09	GB	BE
Amazon Web Services	16509	3.34.51.207	2021-01-01	CN	KR
Amazon Web Services	16509	34.220.187.142	2020-12-20	CN	US
Digital Ocean	14061	165.22.79.71	2022-12-27	IR	DE
Digital Ocean	14061	159.203.41.88	2022-11-26	IR	CA
Digital Ocean	14061	134.209.80.96	2022-11-03	IR	NL

Table 4 shows the final numbers of IP addresses in the dataset from January 2015 to December 2022. The increase in the numbers per year is the result of new devices joining the Atlas. Some devices were also disconnected from the Atlas, which decreased the numbers. In total, 2,913 days are included in the dataset. Several daily reports are missing in the Atlas repository: 1 day in 2016 (2016-05-03), 2 days in 2018 (2018-02-19, 2018-06-29), and 4 days in 2019 (2019-02-01, 2019-02-02, 2019-02-03, 2019-12-24). Two reports were excluded due to corrupted JSON files: 1 day in 2018 (2018-02-21) and 1 day in 2022 (2022-01-21).

**Table 4:** Number of ground-truth IP addresses in the historical dataset.

2015	2016	2017	2018	2019	2020	2021	2022	Total
148,060	148,935	173,145	164,917	149,272	159,722	155,075	141,835	1,240,961

IPv6 addresses are not included in the historical dataset. Their numbers were not sufficient to present firm results, which is a necessity in forensics. This could change in the future as the number of Atlas devices with IPv6 addresses increases.

Algorithm 1 shows the pseudocode to compile the historical dataset. The input data come from the RIPE Atlas archive. The algorithm output is the historical dataset that allows for a transparent assessment of the evidential value of IP country location. Line no. 2 shows the processing of the archive file for the past day. The file contains operational data of the Atlas devices. The relevant data are filtered on line no. 3. Lines no. 4 and 5 show the processing to exclude data that would lead to wrong results. Specifically, devices that were not active on the past day and devices with invalid data are removed. Line no. 6 deals with the validation of IP addresses. The last step of the loop processes the daily data in historical order. Line no. 9 shows the processing of the entire history to avoid multiplication of the results obtained from the same addresses. The most distant device addresses are used. Line no. 10 deals with the removal of unreliable devices. Line no. 11 filters the IP addresses that are irrelevant for the evidential value. These addresses are frequently used in multiple countries and owned by cloud service providers. Finally, line no. 12 deals with the verification that the number of IP addresses in a country and their geographical distribution are suitable to assess the evidential value.

 
_______________________ 
Algorithm 1 Pseudocode to compile historical dataset to assess evidential value 
of IP country location___________________________________________________________________________ 
Require: RIPE Atlas archive ftp.ripe.net/ripe/atlas/probes/archive/ 
Ensure: Valid historical dataset 
  1:  for day in history do 
 2:       Read archive file for day 
 3:       Decompress, filter, and convert day data 
  4:       Remove day devices not connected 
  5:       Remove day devices with invalid data 
  6:       Validate day IP addresses 
  7:       Link day data to history 
 8:  end for 
 9:  Filter history IP addresses repeating in a row of days 
10:  Filter history unreliable devices 
11:  Filter history irrelevant IP addresses 
12:  Use history properties to check its validity in country__________________________    

## Evidential Value Assessment

**Figure 4:**
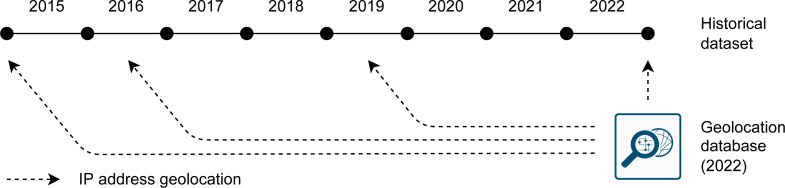
Geolocation of IP addresses using the database dated to the end of the compiled historical dataset.

IP addresses in the compiled historical dataset were located using a geolocation database, as shown in Fig. 4. IP2Location (2022b) country database was used, which is available in the commercial (DB1) and free version (DB1 LITE). The commercial version is supposed to provide better accuracy. On the other hand, the commercial version cannot be redistributed with the source code to apply the data to future evidence. For this reason, it is explicitly stated which database version was used for the particular result, and the use of the database versions is further discussed. The build date of the commercial version is Aug 26th 2022. The free version was released on Aug 27th 2022. The dates were obtained from the binary data encoded in the database binary file. Therefore, any misinterpretation of the date obtained from the filename or filesystem metadata was avoided. The database dates are within one year of the end of the historical dataset (December 2022). The end date of the historical range can be exactly aligned with the particular date of the evidence. The geolocation database can also be (approximately) dated to the date of the evidence. A total of 252 IP addresses were not located by the commercial version of the database.

 The evidential value is first assessed by the naive methods. These methods are shown to give unreliable results. The evidential method is next used to assess the evidential value.

### Naive methods

Three naive methods were elaborated: (i) global accuracy, (ii) single observation, and (iii) cross country. The naive methods are applied to Iran with 33,125 IP addresses in the historical dataset, and Ukraine with 11,345 IP addresses. These countries were selected as the naive methods give misleading results and are excluded by the evidential method. Table 5 shows the number of IP addresses per year. All IP address coordinates were verified to be within the countries to exclude the coordinate and country mismatch.

**Table 5:** Dataset of IP addresses in countries with the naive methods applied.

Country	2015	2016	2017	2018	2019	2020	2021	2022	Total
IR	1,457	2,159	2,918	5,337	5,650	5,561	5,539	4,504	33,125
UA	1,740	1,526	1,344	1,518	1,815	1,726	739	937	11,345

**Figure 5:**
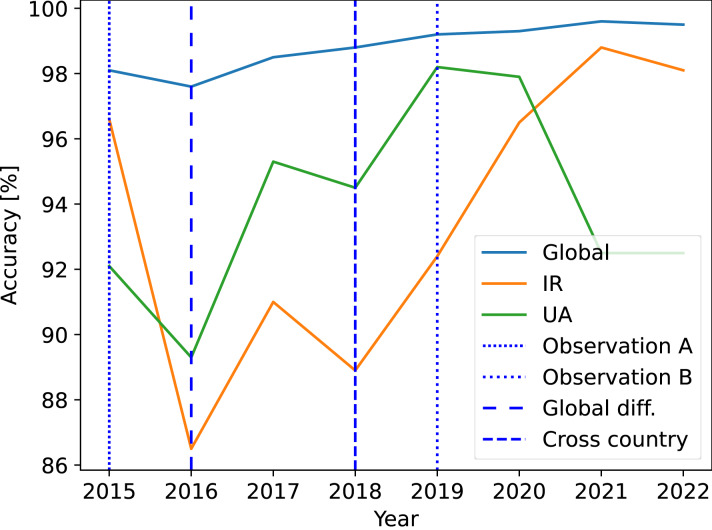
Misleading results obtained by the naive methods. Commercial version of the location database was used.

The first method is a straightforward approach that applies the global accuracy to a country. However, this leads to inaccurate results, as there can be a large difference between global and country-specific accuracy. Figure 5 shows an example of this difference. The global accuracy in 2016 was 97.6%. If we apply this accuracy to the evidence that a device (user) was in Iran on the given date in 2016, we obtain a false evidential value of the claim. The accuracy of IP country location in Iran in 2016 was 86.5%, which is not acceptable to be used as evidence (depending on the case). The method of single accuracy observation can also lead to an inaccurate result. For example, the country location accuracy in Ukraine in 2019 was 98.2% as shown in Fig. 5 for observation B. If this value is used as the evidential value for the evidence that a device (user) was in Ukraine on the given day in 2015, the evidential value is incorrect. The country location accuracy in Ukraine in 2015 was 92.1% as shown in Fig. 5 for observation A. The problem of the third naive method, which is the accuracy application across countries, is shown for the year 2018. In this year, the accuracy of IP country location in Iran was 88.9% and in Ukraine 94.5%, which means a difference of approx. 5% between the countries. Therefore, it is questionable whether the accuracy can be applied across countries. All accuracy values were obtained using the commercial version of the geolocation database.

The evidential method will not accept these countries for location evidence dated to 2022. The historical minimal accuracy values were 86.5% and 89.3%, and the overall accuracy was below the 95th percentile. The ground-truth IP addresses were also inappropriately distributed in the countries, which is discussed further.

### Evidential method

The evidential method is universal and can be applied to any country provided the number of the ground-truth IP addresses and their historical coverage is sufficient. The particular country validity to derive the evidential value of the IP country location evidence is to be determined by the expert witness. Each evidence has a different purpose and relationship to other evidence. The validity of the specific country is further discussed in the section ‘Method and data validity’. Seven countries were selected in order to demonstrate the application of the method to country data. These countries were selected according to several criteria, which included (i) the number of IP addresses in the historical dataset, (ii) geography without major exclaves and enclaves, and (iii) the population not present in a limited area. The results presented for the countries have no relation to those for other countries. This is also valid for larger geographical regions, such as the regions of Europe (West, East, Central), and smaller regions, such as US states.

Table 6 shows the number of IP addresses per year in the historical dataset for the selected countries, which are Germany, France, Italy, Austria, United States, Great Britain, and India. All IP address coordinates archived by the Atlas were verified to be within the countries in order to exclude coordinate and country mismatch. The Metropolitan (European) part of France is used and the IP addresses from France overseas regions are excluded. In general, the evidential value of IP country location may not be processed when country exclaves, including overseas regions, are the subject of location evidence. The list of countries in Table 6 includes the US, which is processed as one country. The US states may be considered individually, which is also valid for other countries that are divided into semi-independent (from the networking point of view) larger regions. The reason is that the country-related problems can also be applicable to the US states. In particular, it is the presence of public (IXP—Internet Exchange Point) or private peering points in the individual states. The presence of global Internet service providers in the states also plays an important role in the IP country/state location as discussed in the section ‘Problem and contribution’. The geography of the US states (*e.g.*, in-land, number of neighboring states) also has an effect on the accuracy of the IP country/state location.

**Table 6:** Dataset of IP addresses in countries with the evidential method applied.

Country	2015	2016	2017	2018	2019	2020	2021	2022	Total
DE	51,752	57,376	80,010	79,136	74,314	78,636	78,169	66,181	565,574
FR	10,567	6,349	7,114	5,763	5,922	7,222	5,100	5,073	53,110
IT	4,273	4,204	6,061	5,052	3,723	4,182	5,678	6,371	39,544
AT	4,228	6,905	6,674	4,156	1,615	1,434	1,841	1,941	28,794
US	4,866	3,727	3,624	3,520	3,364	3,144	3,458	4,627	30,330
GB	5,056	3,788	3,668	2,843	2,332	2,869	2,850	2,518	25,924
IN	653	2,214	2,177	2,800	2,497	5,340	5,987	4,796	26,464

**Figure 6:**
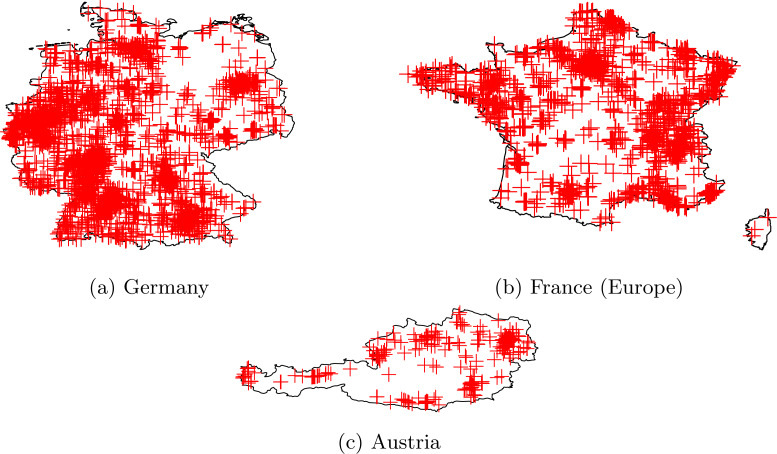
Geographical coverage of the historical dataset in countries. The uniform coverage in a country is not crucial. A cross may represent more devices with all previous IP addresses used. Maps are not in scale.

Figure 6 shows the geographical coverage of the historical dataset in the countries. Cases (a) and (b) show a dense coverage in Germany and France. Case (c) shows Austria with some sparsely covered areas. It should be noted that the even geographical coverage of the ground-truth IP addresses is not crucial. In any country, there are densely and sparsely populated areas, such as the mountain area in Austria. In dense places, more IP network segments are used, and these segments should be represented in the dataset. Note that a single cross on the map may represent more Atlas devices and all their previous IP addresses. Coverage maps for other countries are available at Komosny (2023).

Table 7 shows the accuracy of IP country location obtained using the historical data. The accuracy was greater than 99% in DE, AT, US, GB, and IN. The accuracy was greater than 98% in France. The lowest accuracy was observed in Italy, around 97.5%. There were no significant differences between the commercial and free database versions. It should be noted that the difference may be significant in other countries and also in the same countries for cellular IP addresses. There were some nonlocated IP addresses in the countries for both database versions. Most of the nonlocated IP addresses belonged to address ranges dedicated for special purposes, such as ‘APNIC Research and Development’.

**Table 7:** Accuracy of IP country location in countries. Historical data of eight years were used. The difference between the database versions may be significant in other countries.

Database	DE	FR	IT	AT	US	GB	IN
Commercial (DB1)	99.64	98.74	97.41	99.57	99.39	99.43	99.79
Free (DB1 LITE)	99.63	98.71	97.4	99.56	99.42	99.39	99.79

**Figure 7:**
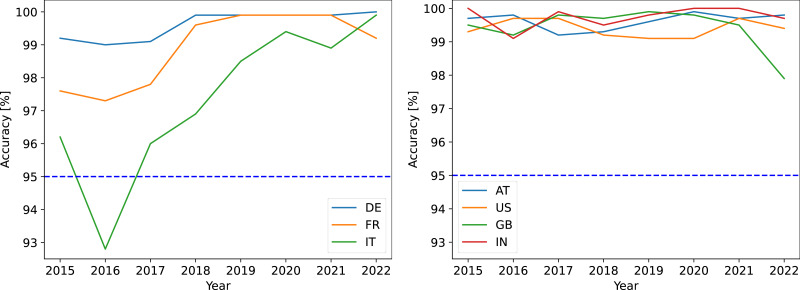
Changes in the accuracy of IP country location. Commercial version of the location database was used.

Figure 7 shows the changes in accuracy over the years. Relatively consistent accuracy was observed in the countries. The largest changes occurred in Italy, with a minimal value of 92.8% in 2016. Similar results were observed for the commercial and free versions of the database. The results show that it is unclear whether several previous databases should be used instead of a single database. The reason is that the accuracy does not constantly increase towards the date of the database release.

Table 8 shows the evidential data of IP country location evidence dated to 2022 in the countries. The column ‘Hist min’ shows the minimal yearly value of the location accuracy. The column ‘Hist all’ shows the accuracy obtained for all years in the historical dataset. The range of historical data is to be determined by the expert witness (eight years were used in this case). The column ‘Addresses’ shows the total number of the ground-truth IP addresses in the country. The column ‘Valid geography’ refers to the appropriate geographical distribution of the ground-truth IP addresses. The column ‘Valid evidence’ refers to whether the IP country location evidence can be used. It should be noted that this statement may not be generalized. Each evidence should be processed individually, and the purpose of the evidence should also be considered. The historical accuracy below the 95th percentile may be the reason to deny the IP country location evidence in Italy.

**Table 8:** Evidential data of IP country location evidence dated to 2022. Note that each evidence should be processed individually.

Country	Hist min	Hist all	Addresses	Valid geography	Valid evidence
IR	86.5	94.1	33,125	NO	NO
UA	89.3	94.4	11,345	NO	NO
DE	99.0	99.6	565,574	YES	YES
FR	97.3	98.7	53,110	YES	YES
IT	92.8	97.4	39,544	YES	NO
AT	99.2	99.6	28,794	YES	YES
US	99.1	99.4	30,330	YES	YES
GB	97.9	99.4	25,924	YES	YES
IN	99.1	99.8	26,464	YES	YES

## Tampered Evidence

**Figure 8:**
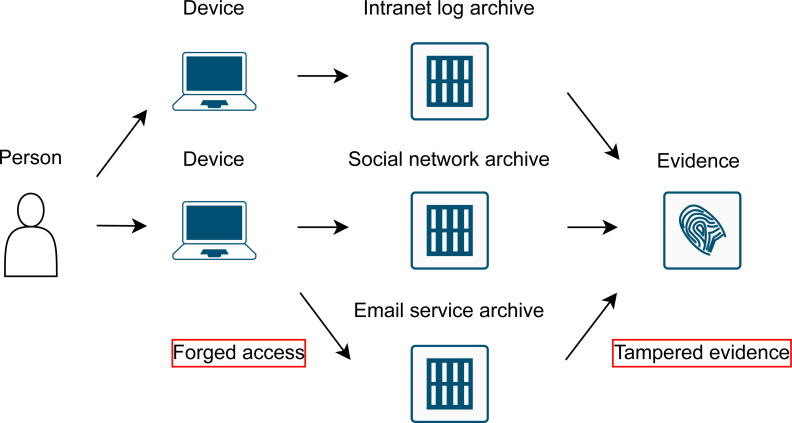
Location evidence obtained from IP geolocation; source data are provided from multiple online services. The person used the services in a short period of time. Any inconsistency leaves the evidence of the IP country location void.

Any evidence can be tampered. IP geolocation evidence is difficult to manipulate, as the IP addresses are stored at the service providers and they are not accessible by the person. There is a possibility that someone can set up forged access to a service. Such access can be implemented by sharing the credentials (username, password) with other person(s) and asking them to use the service at the given time. Additionally, a VPN/proxy can be used to create a service log entry that points to the different country. However, such situations can be inspected. People typically use many online services, and such tampered access would need to be done across all the services in question. An empirical list of common online services is given in Table 1. Any inconsistency between the services should be considered, as shown in Fig. 8. The figure shows three services: company intranet, social network application, and email. Different countries used to access these services in a short period of time would leave the country location evidence void.

**Figure 9:**
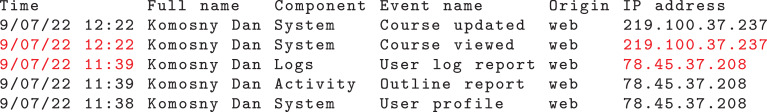
Example of log entries in the elearning system. Changed IP addresses point to countries between which it is not possible to travel during the time difference of the records.

It is also possible to detect tampered evidence or credentials sharing using a single service log. An impossible change between IP addresses can be found by a time analysis. The time analysis can include any user action (*e.g.*, document view, form update, page change) and not only the login events. An example is shown in Fig. 9. The figure shows sample log entries of an educational service(elearning) that provides access to course curriculum, online exams, teacher’s notes, and other data. The system records the time of user actions and the IP addresses used, along with other information, including the full student name and the description of the event, Moodle (2022). The figure shows a situation where IP addresses changed in a short period of time, which was caused by the use of a VPN service (http://www.vpngate.net). In this example, the country was changed from the Czech Republic (true country) to Japan (false country). The time between logged records can be used to determine whether it is possible for a person to travel this distance.

## Method and Data Validity

There are several important prerequisites for the validity of the method and the results. Generally, one cannot assume that a location accuracy result obtained some time ago is applicable to the current location evidence. It also holds for the case where the location evidence on a past date is being assessed using the accuracy data that come from a more distant past date. The reason is that the accuracy of IP geolocation changes over time. Therefore, the historical data should match the date of the location evidence. The number of ground-truth IP addresses and the range of historical data is to be determined by the expert witness. It is the authority of the expert witness to conclude that the location evidence obtained from IP country geolocation is valid for a specific country.

The results presented are valid only for the countries listed, and they should not be applied to other countries. The reason is that the countries differ in ICT, population density, and geography. In terms of the country geography, the presence of nonstandard shapes, *e.g.*, exclaves/enclaves, makes the IP country location accuracy specific. The number of neighboring countries is also important. The locations used were obtained from the IP2Location country geolocation database, both commercial and free versions. Therefore, the results are valid for these databases.

The evidential method is based on the IP addresses that are used by end devices, which belong to RIPE Atlas. These devices are connected to organization and home LANs. For this reason, the results are not valid for IP addresses that are used by cellular devices. The results are valid for devices that connect *via* WiFi, as their IP address is obtained from a LAN (DHCP server). The Atlas devices are distributed by RIPE NCC for free to persons who operate them. The aim is to build the largest Internet measurement network in the world. The distribution of the devices is controlled by RIPE to achieve an even coverage across the IP address space(autonomous systems). This is how the large number of devices and their broad coverage are maintained (RIPE NCC, 2022a). The person operating a device sets its location in the form of country and GPS coordinates. This location is used in Internet geographical-based measurements, which is the main purpose of the Atlas. It is possible that a person specifies the wrong country for a device. Individual devices with a wrong country set would decrease the IP geolocation accuracy at the country level and not improve it. Therefore, the numbers presented can be considered as the worst-case values.

In the future, the method can also be extended to process the evidential value of IP city location. The Atlas devices provide the locations in the form of geographical coordinates. The city of the device can be obtained using reverse geocoding. However, the accuracy of IP geolocation at the city level is currently low, making it unusable in forensics. City-level IP geolocation may be more accurate in the future due to technological progress. For example, more sources of geographical landmarks are available to populate geolocation databases, such as web cameras with their IP address and location known, Li et al. (2021). In addition, the number of locations reported by smartphone users will probably grow as more free applications are available. Such applications report the location of the device along with its IP address in exchange for free use of the application (provided that the user grants permission), as discussed in detail in the related work. These crowd-sourced location data are used to populate geolocation databases and thus improve their accuracy. On the other hand, the accuracy of IP address location will always be limited by the movement in the IP address space due to their reallocations. In particular, IP addresses of mobile devices are known to be frequently reallocated to locations, which are separated by large distances.

## Conclusion

A solution was introduced to assess the evidential value of IP country location. Global and country-specific accuracy should not be applied uniformly, as each country is different in ICT, geography, and demography. Furthermore, the accuracy of IP geolocation is dynamic due to movements in the IP address space. Therefore, single accuracy observations should not be trusted. There is also a lack of free and constantly updated ground-truth data to be used in forensics. The presented solution addresses these problems. The evidential data were given for several countries. It was also shown that naive methods give results that may lead to wrong evidential values. The evidential method excludes such misleading results.

The presented evidential data may not be used generally. Each evidence should be processed individually, and the purpose of the evidence should also be considered. For example, it is up to the expert witness to consider the removal of IP addresses from the dataset that belong to cloud service providers. The results presented are also valid only for the countries listed, and they cannot be applied to other countries. This also holds for the geolocation database versions (free and commercial) as the difference between the database versions may be significant for other countries. Historical data should be updated to match the date of the evidence. The source code to replicate the results is available at Komosny (2023).

Future work after several years may cover the evidential value of country geolocation of IPv6 addresses. In this work, IPv6 addresses were not included due to their insufficient number to provide firm results, which is a necessity in forensics. The list of countries that demonstrate the application of the method can be extended as more ground-truth IP addresses will be available. In the future, the method could also be used to derive the evidential value of IP geolocation at the city level. The current accuracy of the IP city location is low, making it unusable in forensics. Future technological progress could improve it to a sufficient level. However, there will still be problems present, which were described in the article. For example, there are constant changes in the IP address space due to address reallocations. In particular, cellular devices are known to change their IP address frequently, which is detrimental to the accuracy of IP geolocation using geolocation databases.

## Additional Information and Declarations

### Data Availability

The following information was supplied regarding data availability: The source code and raw data are available at GitHub: https://github.com/komosny/IP-country-forensic-evidence (v1.0.1) and Zenodo: https://doi.org/10.5281/zenodo.7583617. This work used IP2Location LITE data available from the IP2Location™ LITE IP-COUNTRY Database: https://lite.ip2location.com/database/ip-country.
